# New progress on the operator inequalities involving improved Young’s and its reverse inequalities relating to the Kantorovich constant

**DOI:** 10.1186/s13660-017-1344-9

**Published:** 2017-04-05

**Authors:** Jie Zhang, Junliang Wu

**Affiliations:** College of Mathematics and Statistics, Chongqing University, Chongqing, 401331 China

**Keywords:** 15A15, 15A42, 15A60, 47A30, Young’s inequality, Kittaneh-Manasrah inequality, Kantorovich constant, operator inequality, progress

## Abstract

The purpose of this paper is to give a survey of the progress, advantages and limitations of various operator inequalities involving improved Young’s and its reverse inequalities related to the Kittaneh-Manasrah inequality. We also present our new progress to the related research topics. New scalar versions of Young’s inequalities are promoted, the operator version and the Hilbert-Schmidt form also get a promotion.

## Introduction

As is well known, the famous Young’s inequality for real numbers is that

1.1 $$ (1 - u)a + ub \ge a^{1 - u}b^{u},\quad\mbox{where } a,b > 0, u \in [ 0,1 ] $$

which is called the *u*-weighted arithmetic-geometric mean inequality.

Recently, research in this area has received continuous attention. Many researches have spent lots of energy on it due to the applications in various fields. In [1, 2], Kittaneh and Manasrah researched Young’s inequality and obtained the following results:

1.2 $$ a^{1 - u}b^{u} + r ( \sqrt{a} - \sqrt{b} )^{2} \le ( 1 - u )a + ub \le a^{1 - u}b^{u} + s ( \sqrt{a} - \sqrt{b} )^{2}, $$

where $a,b > 0$, $u \in [ 0,1 ]$ and $r = \min \{ 1 - u,u \}$, $s = \max \{ 1 - u,u \}$.

The left inequality of (1.2) can be regarded as a refinement of Young’s inequality and the right one can be regarded as a reverse of Young’s inequality.

In [3], Zhao and Wu deepened inequality (1.2) and showed another refinement of Young’s inequality as follows:

If $0 \le u \le\frac{1}{2}$, then

1.3 $$ ( 1 - u )a + ub \ge a^{1 - u}b^{u} + u ( \sqrt{a} - \sqrt{b} )^{2} + r_{0} \bigl( \sqrt[4]{ab} - \sqrt{a} \bigr)^{2} ; $$

If $\frac{1}{2} < u \le1$, then

1.4 $$ ( 1 - u )a + ub \ge a^{1 - u}b^{u} + ( 1 - u ) ( \sqrt{a} - \sqrt{b} )^{2} + r_{0} \bigl( \sqrt[4]{ab} - \sqrt{b} \bigr)^{2} , $$

where $a,b \ge0$, $u \in [ 0,1 ]$ and $r = \min \{ u,1 - u \}$, $r_{0} = \min \{ 2r,1 - 2r \}$.

Zhao and Wu also obtained a more precise form of the reverse Young’s inequality as follows:

If $0 \le u \le\frac{1}{2} $, then

1.5 $$ ( 1 - u )a + ub \le a^{1 - u}b^{u} + ( 1 - u ) (\sqrt{a} - \sqrt{b} )^{2} - r_{0} \bigl( \sqrt[4]{ab} - \sqrt{b} \bigr)^{2} ; $$

If $\frac{1}{2} < u \le1 $, then

1.6 $$ ( 1 - u )a + ub \le a^{1 - u}b^{u} + u(\sqrt{a} - \sqrt{b} )^{2} - r_{0} \bigl( \sqrt[4]{ab} - \sqrt{a} \bigr)^{2} , $$

where $r = \min \{ u,1 - u \}$, $r_{0} = \min \{ 2r,1 - 2r \}$ and $a,b \ge0$, $u \in [ 0,1 ]$.

In [4], Furuichi obtained Young’s inequality about Specht’s ratio as follows:

1.7 $$ ( 1 - u )a + ub \ge S \bigl( h^{r} \bigr)a^{1 - u}b^{u}, $$

where $r = \min \{ 1 - u,u \}$, $h = \frac{b}{a}$, and $a,b > 0$, $u \in [ 0,1 ]$.

In [5], Tominaga got the reverse Young inequality with the help of Specht’s ratio. He gave the following inequality:

$$( 1 - u )a + ub \le S ( h )a^{1 - u}b^{u}. $$

Article [6] pointed out that Specht’s ratio and the Kantorovich constant have the relationship as follows:

1.8 $$ S \bigl( t^{r} \bigr) \le K ( t,2 )^{r}, $$

where $t > 0$ and $0 \le r \le\frac{1}{2}$.

Based on this idea, in the article [6], the authors got the refinement of Young’s inequality:

1.9 $$ ( 1 - {u} )a + ub \ge K(h,2)^{r}a^{1 - u}b^{u}, $$

where $r = \min \{ 1 - u,u \}$, $h = \frac{b}{a}$, and $a,b \ge0$, $u \in [ 0,1 ]$.

Generally, the Kantorovich constant is written as $K ( {t},2 ) = \frac{(t + 1)^{2}}{4t}$ for ${t} > 0$, which has properties $K ( t,2 ) = K ( \frac{1}{t},2 ) \ge 1$ ($t > 0 $), and $K ( t,2 )$ is increasing on $[1,\infty)$ and is decreasing on $( 0,1 )$.

In [7], Zhao and Wu made a further study about inequality (1.2) with the Kantorovich constant and gave the following results:

1.10 $$ ( 1 - {u} )a + ub \ge K ( \sqrt{h},2 )^{r'}a^{1 - u}b^{u} + r ( \sqrt{a} - \sqrt{b} )^{2}, $$

where $r = \min \{ u, 1 - u \}$, $r' = \min \{ 2r,1 - 2r \}$, $a,b > 0$, $u \in [ 0,1 ] - \{ \frac{1}{2} \}$ and $h = \frac{b}{a}$.

And then Zhao and Wu made a reverse refinement of the second inequality in (1.2)

1.11 $$ ( 1 - {u} )a + ub \le K ( \sqrt{h},2 )^{ - r'}a^{1 - u}b^{u} + s ( \sqrt{a} - \sqrt{b} )^{2}, $$

where $a,b > 0$, $u \in [ 0,1 ] - \{ \frac{1}{2} \} $, $h =\frac{b}{a}$ and $r = \min \{ u, 1 - u \}$, $r' = \min \{ 2r,1 - 2r \}$, $s = \max \{ u, 1 - u \}$.

Let us take a closer look at [8] where Liao et al. made a reverse refinement for Young’s inequality as follows:

1.12 $$\begin{aligned}& ( 1 - u )a + ub \le K ( h,2 )^{R}a^{1 - u}b^{u}, \end{aligned}$$

1.13 $$\begin{aligned}& ( 1 - u )a + ub \le r ( \sqrt{a} - \sqrt{b} )^{2} + K ( \sqrt{h},2 )^{R'}a^{1 - u}b^{u}, \end{aligned}$$

where $r = \min \{ 1 - u,u \}$, $R' = \max \{ 2r,1 - 2r \}$, $R = \max \{1 - u,u \}$ and $a,b > 0$, $u \in [ 0,1 ]$, $h = \frac{b}{a}$.

In [9], Liao and Wu further deepened the results of inequality (1.10) and obtained the following results:

If $0 < u \le\frac{1}{2}$, then

1.14 $$ ( 1 - u )a + ub \ge u ( \sqrt{a} - \sqrt{b} )^{2} + r_{0} \bigl( \sqrt[4]{ab} - \sqrt{a} \bigr)^{2} + K \bigl( \sqrt[4]{h},2 \bigr)^{r_{1}}a^{1 - u}b^{u}; $$

If $\frac{1}{2} < u < 1$, then

1.15 $$ ( 1 - u )a + ub \ge ( 1 - u ) ( \sqrt{a} - \sqrt{b} )^{2} + r_{0} \bigl( \sqrt[4]{ab} - \sqrt{b} \bigr)^{2} + K \bigl( \sqrt[4]{h},2 \bigr)^{r_{1}}a^{1 - u}b^{u}, $$

where $h = \frac{b}{a}$, $r = \min \{ u,1 - u \}$, $r_{0} = \min \{ 2r,1 - 2r \}$, $r_{1} = \min \{ 2r_{0},1 - 2r_{0} \}$.

Similarly, Liao and Wu deepened the reverse results of inequality (1.10) as follows:

If $0 < u \le\frac{1}{2}$, then

1.16 $$ ( 1 - u )a + ub \le ( 1 - u ) ( \sqrt{a} - \sqrt{b} )^{2} - r_{0} \bigl( \sqrt[4]{ab} - \sqrt{b} \bigr)^{2} + K \bigl( \sqrt[4]{h},2 \bigr)^{ - r_{1}}a^{1 - u}b^{u}; $$

If $\frac{1}{2} < u < 1$, then

1.17 $$ ( 1 - u )a + ub \le u ( \sqrt{a} - \sqrt{b} )^{2} - r_{0} \bigl( \sqrt[4]{ab} - \sqrt{a} \bigr)^{2} + K \bigl( \sqrt[4]{h},2 \bigr)^{ - r_{1}}a^{1 - u}b^{u}. $$

In [10], Hizrallah and Kittaneh showed a square form of the refinement of the first inequality of (1.2) as follows:

1.18 $$ \bigl( ( 1 - u )a + ub \bigr)^{2} \ge \bigl( a^{1 - u}b^{u} \bigr)^{2} + r^{2} ( a - b )^{2}, $$

where $a,b > 0$, $u \in [ 0,1 ]$ and $r = \min \{ u,1 - u \}$.

In [11] He and Zou presented a reverse of the second inequality of (1.2) as follows:

1.19 $$ \bigl( ( 1 - u )a + ub \bigr)^{2} \le \bigl( a^{1 - u}b^{u} \bigr)^{2} + s^{2} ( a - b )^{2}, $$

where $a,b > 0$, $u \in [ 0,1 ]$ and $s = \max \{ u, 1 - u \}$.

For the sake of convenience, we have to introduce a set of symbols. Let $A,B \in B ( H )$ be two positive and invertible operators, where $B ( H )$ is regarded as the set of all bounded linear operators on a complex separable Hilbert space *H*. $A^{*}$ denotes the conjugate operator of *A*. Let $M_{n}$ be the set of $n \times n$ complex matrices. For $A = ( a_{ij} ) \in M_{n}$, the Hilbert-Schmidt norm of *A* is defined by $\Vert A \Vert _{2} = \sqrt{\sum_{i,j = 1}^{n} a_{ij}^{2}}$, $\Vert \ \Vert _{2}$ has the unitarily invariant property, that is, $\Vert UAV \Vert _{2} = \Vert A \Vert _{2}$ for all $A \in M_{n}$ and all unitary matrices $U,V \in M_{n}$. We use the following notations:

$$\begin{aligned}& A\nabla_{{u}}B = ( 1 - {u} )A + {uB}, \\& A\#_{{u}}B = A^{\frac{1}{2}} \bigl( A^{ - \frac{1}{2}}BA^{ - \frac{1}{2}} \bigr)^{{u}}A^{\frac{1}{2}}, \\& A!_{u}B = \bigl( ( 1 - u )A^{ - 1} + uB^{ - 1} \bigr)^{ - 1}. \end{aligned}$$

The operator versions $A\nabla_{{u}}B$, $A\#_{{u}}B$, $A!_{{u}}B$ are called the arithmetic mean, geometric mean and harmonic mean, respectively. When $u = \frac{1}{2}$, these operators can be written by simplification as $A\nabla B$, $A\# B$, $A!B$.

The article [12] presented the matrix version of (1.1). For any $B,C \in M_{n}$, if *B* is a positive definite matrix and *C* is an invertible matrix, then

1.20 $$ A\nabla_{u}B \ge C^{*} \bigl( \bigl( C^{*} \bigr)^{ - 1}BC^{ - 1} \bigr)^{ - u}C, $$

where $A = CC^{*}$ and $u \in [ 0,1 ]$.

Specially, if $C = A^{\frac{1}{2}}$, then $C^{*} ( ( C^{*} )^{ - 1}BC^{ - 1} )^{ - u}C = A\#_{u}B$ and inequality (1.20) can be written as $A\nabla_{u}B \ge A\#_{u}B$.

Next, we have to introduce the development of operator inequalities. In [2], Kittaneh and Manasrah took a step further and got the matrix version of inequality (1.2)

1.21 $$\begin{aligned} 2r \bigl( A\nabla B - C^{*} \bigl( \bigl( C^{*} \bigr)^{ - 1}BC^{ - 1} \bigr)^{ - \frac{1}{2}}C \bigr) \le& A \nabla_{u}B - C^{*} \bigl( \bigl( C^{*} \bigr)^{ - 1}BC^{ - 1} \bigr)^{ - u}C \\ \le&2s \bigl( A\nabla B - C^{*} \bigl( \bigl( C^{*} \bigr)^{ - 1}BC^{ - 1} \bigr)^{ - \frac{1}{2}}C \bigr), \end{aligned}$$

where $u \in [ 0,1 ]$, $r = \min \{ u,1 - u \}$ and $s = \max \{ u,1 - u \}$.

Based on the idea, articles [7, 8] and [9] also got the corresponding operator forms of inequality (1.10)-(1.19).

In [10], inequality (1.18) for the Hilbert-Schmidt norm form was obtained by Hirzallah and Kittaneh, that is, if $A,B,X \in M_{n}$, where *A*, *B* are positive semi-definite matrices, then

1.22 $$ \bigl\Vert ( 1 - u )AX + uXB \bigr\Vert _{2}^{2} \ge \bigl\Vert A^{1 - u}XB^{u} \bigr\Vert _{2}^{2} + r^{2} \Vert AX - XB \Vert _{2}^{2}, $$

where $u \in [ 0,1 ]$ and $r = \min \{ u,1 - u \}$.

The reverse inequality (1.19) for the Hilbert-Schmidt norm form was obtained by Kittaneh and Manasrah in [2] as the following result:

1.23 $$ \bigl\Vert ( 1 - u )AX + uXB \bigr\Vert _{2}^{2} \le \bigl\Vert A^{1 - u}XB^{u} \bigr\Vert _{2}^{2} + s^{2} \Vert AX - XB \Vert _{2}^{2}, $$

where $u \in [ 0,1 ]$ and $s = \max \{ u,1 - u \}$.

We find that there are still limitations in the previous inequalities. This paper continues to research the refinement of Young’s inequality. The difference is that we present Young’s inequalities in four more precise intervals of $[0,1]$ other than two sections of $[0,1]$, and also the operator form and the matrix version get a promotion.

In Section 2, a new scalar version of Young’s inequality and its reverse with the Kantorovich constant are first given. In Section 3, we obtain the corresponding new operator inequalities on the Hilbert space. Finally, in Section 4, we mainly show the matrix version of inequalities for the Hilbert-Schmidt norm.

## New progress of Young’s and its reverse inequalities

In this section, we mainly present the improved scalar Young and its reverse inequalities relating to the Kantorovich constant.

### Theorem 2.1

*Let* $a,b > 0$, $u \in ( 0, 1 )$, *we have*

1. *If* $0 < {u} < \frac{1}{4}$, *then*
  2.1 $$\begin{aligned} ( 1 - u )a + ub \ge& u ( \sqrt{a} - \sqrt{b} )^{2} + 2u \bigl( \sqrt[4]{ab} - \sqrt{a} \bigr)^{2} + r \bigl( \sqrt{a} - \sqrt[8]{a^{3}b} \bigr)^{2} \\ &{}+ K \bigl( \sqrt[8]{h},2 \bigr)^{r_{1}}a^{1 - u}b^{u}, \end{aligned}$$
  *where* $h = \frac{b}{a}$, $r = \min \{ 1 - 4u,4u \}$, $r_{1} = \min \{ 2r,1 - 2r \}$.
2. *If* $\frac{1}{4} \le u < \frac{1}{2}$, *then*
  2.2 $$\begin{aligned} ( 1 - u )a + ub \ge& u ( \sqrt{a} - \sqrt{b} )^{2} + ( 1 - 2u ) \bigl( \sqrt[4]{ab} - \sqrt{a} \bigr)^{2} + r \bigl( \sqrt[4]{ab} - \sqrt[8]{a^{3}b} \bigr)^{2} \\ &{}+ K \bigl( \sqrt[8]{h},2 \bigr)^{r_{1}}a^{1 - u}b^{u}, \end{aligned}$$
  *where* $h = \frac{b}{a}$, $r = \min \{ 2 - 4u,4u - 1 \}$, $r_{1} = \min \{ 2r,1 - 2r \}$.
3. *If* $\frac{1}{2} \le u < \frac{3}{4}$, *then*
  2.3 $$\begin{aligned} ( 1 - u )a + ub \ge& ( 1 - u ) ( \sqrt{a} - \sqrt{b} )^{2} + ( 2u - 1 ) \bigl( \sqrt[4]{ab} - \sqrt{b} \bigr)^{2} + r \bigl( \sqrt[4]{ab} - \sqrt[8]{ab^{3}} \bigr)^{2} \\ &{}+ K \bigl( \sqrt[8]{h},2 \bigr)^{r_{1}}a^{1 - u}b^{u}, \end{aligned}$$
  *where* $h = \frac{b}{a}$, $r = \min \{ 3 - 4u,4u - 2 \}$, $r_{1} = \min \{ 2r,1 - 2r \}$.
4. *If* $\frac{3}{4} \le u < 1$, *then*
  2.4 $$\begin{aligned} ( 1 - u )a + ub \ge& ( 1 - u ) ( \sqrt{a} - \sqrt{b} )^{2} + ( 2 - 2u ) \bigl( \sqrt[4]{ab} - \sqrt{b} \bigr)^{2} + r \bigl( \sqrt{b} - \sqrt[8]{ab^{3}} \bigr)^{2} \\ &{}+ K \bigl( \sqrt[8]{h},2 \bigr)^{r_{1}}a^{1 - u}b^{u}, \end{aligned}$$
  *where* $h = \frac{b}{a}$, $r = \min \{ 4 - 4u,4u - 3 \}$, $r_{1} = \min \{ 2r,1 - 2r \}$.

### Proof

The process of the proof of inequalities (2.3) and (2.4) is similar to that of inequalities (2.1) and (2.2), so we only need to prove inequalities (2.1) and (2.2).

We first consider the case $0 < {u} \le\frac{1}{4}$. By inequality (1.10) and inequality (1.14), we have

$$\begin{aligned}& ( 1 - {u} )a + ub - u ( \sqrt{a} - \sqrt{b} )^{2} - 2u \bigl( \sqrt[4]{ab} - \sqrt{a} \bigr)^{2} \\& \quad = 2u\sqrt{ab} + ( 1 - 2u )a - 2u \bigl( \sqrt[4]{ab} - \sqrt{a} \bigr)^{2} \\& \quad = ( 1 - 4u )a + 4u\sqrt[4]{a^{3}b} \\& \quad \ge r \bigl( \sqrt{a} - \sqrt[8]{a^{3}b} \bigr) + K \bigl( \sqrt[8]{h},2 \bigr)^{r_{1}}a^{1 - u}b^{u}. \end{aligned}$$

Then we can conclude the inequality

$$\begin{aligned} ( 1 - u )a + ub \ge& u ( \sqrt{a} - \sqrt{b} )^{2} + 2u \bigl( \sqrt[4]{ab} - \sqrt{a} \bigr)^{2} + r \bigl( \sqrt{a} - \sqrt[8]{a^{3}b} \bigr)^{2} \\ &{}+ K \bigl( \sqrt[8]{h},2 \bigr)^{r_{1}}a^{1 - u}b^{u}. \end{aligned}$$

Then, considering the case $\frac{1}{4} \le u < \frac{1}{2}$, we have

$$\begin{aligned}& ( 1 - u )a + ub - u ( \sqrt{a} - \sqrt{b} )^{2} - ( 1 - 2u ) \bigl( \sqrt[4]{ab} - \sqrt{a} \bigr)^{2} \\& \quad = 2u\sqrt{ab} - ( 1 - 2u ) \bigl( \sqrt{ab} - 2\sqrt[4]{a^{3}b} \bigr) \\& \quad = ( 2 - 4u )\sqrt[4]{a^{3}b} + ( 4u - 1 )\sqrt{ab} \\& \quad \ge r \bigl( \sqrt[8]{a^{3}b} - \sqrt[4]{ab} \bigr)^{2} + K \bigl( \sqrt[8]{h},2 \bigr)^{r_{1}}a^{1 - u}b^{u}. \end{aligned}$$

We conclude the inequality

$$\begin{aligned} ( 1 - u )a + ub \ge& u ( \sqrt{a} - \sqrt{b} )^{2} + ( 1 - 2u ) \bigl( \sqrt[4]{ab} - \sqrt{a} \bigr)^{2} + r \bigl( \sqrt[4]{ab} - \sqrt[8]{a^{3}b} \bigr)^{2} \\ &{}+ K \bigl( \sqrt[8]{h},2 \bigr)^{r_{1}}a^{1 - u}b^{u}. \end{aligned}$$

So the proof is completed. □

It is not hard to see that the results above are superior to inequalities (1.14) and (1.15) through more precise subsections.

And the following result is superior to inequalities (1.16) and (1.17).

### Theorem 2.2

*Let* $a,b > 0$, $u \in ( 0,1 )$, *we have the following*.

1. *If* $0 < u < \frac{1}{4}$, *then*
  2.5 $$\begin{aligned} ( 1 - u )a + ub \le&(1 - u) ( \sqrt{a} - \sqrt{b} )^{2} - 2u \bigl( \sqrt[4]{ab} - \sqrt{b} \bigr)^{2} - r \bigl( \sqrt[8]{ab^{3}} - \sqrt{b} \bigr) \\ &{}+ K \bigl( \sqrt[8]{h},2 \bigr)^{ - r_{1}}a^{1 - u}b^{u}, \end{aligned}$$
  *where* $h = \frac{b}{a}$, $r = \min \{ 4u,1 - 4u \}$, $r_{1} = \min \{ 2r,1 - 2r \}$.
2. *If* $\frac{1}{4} \le u < \frac{1}{2}$, *then*
  2.6 $$\begin{aligned} ( 1 - u )a + ub \le&(1 - u) ( \sqrt{a} - \sqrt{b} )^{2} - ( 1 - 2u ) \bigl( \sqrt[4]{ab} - \sqrt{b} \bigr)^{2} - r \bigl( \sqrt[8]{ab^{3}} - \sqrt[4]{ab} \bigr)^{2} \\ &{}+ K \bigl( \sqrt[8]{h},2 \bigr)^{ - r_{1}}a^{1 - u}b^{u}, \end{aligned}$$
  *where* $h = \frac{b}{a}$, $r = \min \{ 2 - 4u,4u - 1 \}$, $r_{1} = \min \{ 2r,1 - 2r \}$.
3. *If* $\frac{1}{2} \le u < \frac{3}{4}$, *then*
  2.7 $$\begin{aligned} ( 1 - u )a + ub \le& u ( \sqrt{a} - \sqrt{b} )^{2} - ( 2u - 1 ) \bigl( \sqrt[4]{ab} - \sqrt{a} \bigr)^{2} - r \bigl( \sqrt[8]{a^{3}b} - \sqrt[4]{ab} \bigr)^{2} \\ &{}+ K \bigl( \sqrt[8]{h},2 \bigr)^{ - r_{1}}a^{1 - u}b^{u}, \end{aligned}$$
  *where* $h = \frac{b}{a}$, $r = \min \{ 3 - 4u,4u - 2 \}$, $r_{1} = \min \{ 2r,1 - 2r \}$.
4. *If* $\frac{3}{4} \le u < 1$, *then*
  2.8 $$\begin{aligned} ( 1 - u )a + ub \le& u ( \sqrt{a} - \sqrt{b} )^{2} - ( 2 - 2u ) \bigl( \sqrt[4]{ab} - \sqrt{a} \bigr)^{2} - r \bigl( \sqrt[8]{a^{3}b} - \sqrt{a} \bigr)^{2} \\ &{}+ K \bigl( \sqrt[8]{h},2 \bigr)^{ - r_{1}}a^{1 - u}b^{u}, \end{aligned}$$
  *where* $h = \frac{b}{a}$, $r = \min \{ 4 - 4u,4u - 3 \}$, $r_{1} = \min \{ 2r,1 - 2r \}$.

### Proof

The process of the proof of inequalities (2.7) and (2.8) is similar to that of inequalities (2.5) and (2.6), so we only need to prove inequalities (2.5) and (2.6).

We consider the first case $0 < u < \frac{1}{4}$. By inequality (1.14) obtained by Liao and Wu, we have

$$\begin{aligned}& K \bigl( \sqrt[8]{h},2 \bigr)^{ - r_{1}}a^{1 - u}b^{u} + ( 1 - u ) ( \sqrt{a} - \sqrt{b} )^{2} - ( 1 - u )a - ub \\& \quad = K \bigl( \sqrt[8]{h},2 \bigr)^{ - r_{1}}a^{1 - u}b^{u} + ( 1 - 2u )b + 2u\sqrt{ab} - 2\sqrt{ab} \\& \quad \ge K \bigl( \sqrt[8]{h},2 \bigr)^{ - r_{1}}a^{1 - u}b^{u} + 2u \bigl( \sqrt[4]{ab} - \sqrt{b} \bigr)^{2} + r \bigl( \sqrt[8]{ab^{3}} - \sqrt{b} \bigr)^{2} \\& \qquad {}+ K \bigl( \sqrt[8]{h},2 \bigr)^{r_{1}}a^{u}b^{1 - u} - 2\sqrt{ab} \\& \quad \ge2u \bigl( \sqrt[4]{ab} - \sqrt{b} \bigr)^{2} + r \bigl( \sqrt[8]{ab^{3}} - \sqrt{b} \bigr)^{2}. \end{aligned}$$

That is to say,

$$\begin{aligned} ( 1 - u )a + ub \le& ( 1 - u ) ( \sqrt{a} - \sqrt{b} )^{2} - 2u \bigl( \sqrt[4]{ab} - \sqrt{b} \bigr)^{2} - r \bigl( \sqrt[8]{ab} - \sqrt{b} \bigr)^{2} \\ &{}+ K \bigl( \sqrt[8]{h},2 \bigr)^{ - r_{1}}a^{1 - u}b^{u}. \end{aligned}$$

So the proof is completed. □

### Remark 2.1

By the property of the Kantorovich constant, the inequalities in Theorems 2.1 and 2.2 are the improved results of Liao and Wu in [9].

Next we are going to deduce another form of reverse ratio Young’s inequality by virtue of inequality (1.13).

### Theorem 2.3

*Let* $a,b > 0$, $u \in ( 0,1 )$.

*If* $0 < u < \frac{1}{2}$, *then*

2.9 $$ ( 1 - u )a + ub \le u ( \sqrt{a} - \sqrt{b} )^{2} + r \bigl( \sqrt{a} - \sqrt[4]{ab} \bigr)^{2} + K \bigl( \sqrt[4]{h},2 \bigr)^{R}a^{1 - u}b^{u}; $$

*If* $\frac{1}{2} \le u < 1$, *then*

2.10 $$ ( 1 - u )a + ub \le ( 1 - u ) ( \sqrt{a} - \sqrt{b} )^{2} + r \bigl( \sqrt{b} - \sqrt[4]{ab} \bigr)^{2} + K \bigl( \sqrt[4]{h},2 \bigr)^{R}a^{1 - u}b^{u}, $$

*where* $h = \frac{b}{a}$, $r = \min \{ 2u,1 - 2u \}$, $R = \max \{2r,1 - 2r \}$.

### Proof

The process of the proof of the second inequality is similar to that of the first one, we only need to investigate the first one. By inequality (1.13), we have

$$\begin{aligned} \begin{aligned} &( 1 - u )a + ub - u ( \sqrt{a} - \sqrt{b} )^{2} \\ &\quad = 2u\sqrt{ab} + ( 1 - 2u )a \\ &\quad \le r \bigl( \sqrt{a} - \sqrt[4]{ab} \bigr)^{2} + K \bigl( \sqrt[4]{h},2 \bigr)^{R}a^{1 - u}b^{u}. \end{aligned} \end{aligned}$$

Then we conclude the inequality

$$( 1 - u )a + ub \le u ( \sqrt{a} - \sqrt{b} )^{2} + r \bigl( \sqrt{a} - \sqrt[4]{ab} \bigr)^{2} + K \bigl( \sqrt[4]{h},2 \bigr)^{R}a^{1 - u}b^{u}. $$

So the proof is completed. □

### Remark 2.2

It is worth to mention that we can obtain a new reverse Young’s inequality about Specht’s ratio on the basis of inequalities (2.9) and (2.10). That is:

If $0 < u < \frac{1}{2}$, then

$$( 1 - u )a + ub \le u ( \sqrt{a} - \sqrt{b} )^{2} + r \bigl( \sqrt{a} - \sqrt[4]{ab} \bigr)^{2} + S \bigl( \sqrt[4]{h} \bigr)a^{1 - u}b^{u}. $$

If $\frac{1}{2} \le u < 1$, then

$$( 1 - u )a + ub \le ( 1 - u ) ( \sqrt{a} - \sqrt{b} )^{2} + r \bigl( \sqrt{b} - \sqrt[4]{ab} \bigr)^{2} + S \bigl( \sqrt[4]{h} \bigr)a^{1 - u}b^{u}. $$

When $0 < u < \frac{1}{2}$, it is easy to see that the right-hand side of the inequality above and the corresponding side of the inequality in Theorem 2.3 cannot be compared because the value of $K(\sqrt[4]{h},2)^{R}$ will change with *R*; neither of them is uniformly better than the other. The other case when $\frac{1}{2} \le u < 1$ has the same conclusion analysis, so we omit it here.

### Remark 2.3

If we use $a^{2}$, $b^{2}$ to take the place of *a*, *b* in inequalities (2.9) and (2.10), we can easily obtain the following results:

If $0 < u < \frac{1}{2}$, then

2.11 $$ ( 1 - u )a^{2} + ub^{2} \le u ( a - b )^{2} + r ( a - \sqrt{ab} )^{2} + K ( \sqrt{h},2 )^{R}a^{2 - 2u}b^{2u}; $$

If $\frac{1}{2} \le u < 1$, then

2.12 $$ ( 1 - u )a^{2} + ub^{2} \le ( 1 - u ) ( a - b )^{2} + r ( b - \sqrt{ab} )^{2} + K ( \sqrt{h},2 )^{R}a^{2 - 2u}b^{2u}. $$

Note that we can easily obtain the square form of reverse Young’s inequality on the basis of the above inequalities (2.11) and (2.12) which are more excellent than the inequality in [8].

### Theorem 2.4

*Let* $a,b > 0$, $u \in ( 0,1 )$. *We have the following*.

*If* $0 < u < \frac{1}{2}$, *then*

2.13 $$ \bigl( ( 1 - u )a + ub \bigr)^{2} \le u^{2} ( a - b )^{2} + r ( a - \sqrt{ab} )^{2} + K ( \sqrt{h},2 )^{R}a^{2 - 2u}b^{2u}; $$

*If* $\frac{1}{2} \le u < 1$, *then*

2.14 $$ \bigl( ( 1 - u )a + ub \bigr)^{2} \le ( 1 - u )^{2} ( a - b )^{2} + r ( b - \sqrt{ab} )^{2} + K ( \sqrt{h},2 )^{R}a^{2 - 2u}b^{2u}, $$

*where* $h = \frac{b}{a}$, $r = \min \{ 2u,1 - 2u \}$, $R = \min \{2r,1 - 2r \}$.

### Proof

The process of proving the second inequality is similar to that of proving the first one, so we omit it and only investigate the first one. By inequalities (2.11) and (2.12), we can deduce that

$$\begin{aligned}& \bigl( ( 1 - u )a + ub \bigr)^{2} - u^{2} ( a - b )^{2} \\& \quad = ( 1 - u )a^{2} + ub^{2} - u ( a - b )^{2} \\& \quad \le r ( a - \sqrt{ab} )^{2} + K ( \sqrt{h},2 )^{R} \bigl( a^{1 - u}b^{u} \bigr)^{2}. \end{aligned}$$

And then we can conclude the following inequality:

$$\bigl( ( 1 - u )a + ub \bigr)^{2} \le u^{2} ( a - b )^{2} + r ( a - \sqrt{ab} )^{2} + K ( \sqrt{h},2 )^{R}a^{2 - 2u}b^{2u}. $$

 □

At the end of the section, we are going to consider a new square form based on Theorem 2.3 which is more creative than the inequality in [12].

### Theorem 2.5

*Let* $a,b > 0$, $u \in ( 0,1 )$. *We have the following*.

*If* $0 < u < \frac{1}{2}$, *then*

2.15 $$\begin{aligned} ( a + b )^{2} \le& K ( \sqrt{h},2 )^{R} \bigl( a^{1 - u}b^{u} + a^{u}b^{1 - u} \bigr)^{2} \\ &{}+ r \bigl( ( a - \sqrt{ab} )^{2} + ( b - \sqrt{ab} )^{2} \bigr)+ 2u ( a - b )^{2}; \end{aligned}$$

*If* $\frac{1}{2} \le u < 1$, *then*

2.16 $$\begin{aligned} ( a + b )^{2} \le& K ( \sqrt{h},2 )^{R} \bigl( a^{1 - u}b^{u} + a^{u}b^{1 - u} \bigr)^{2} \\ &{}+ r \bigl( ( a - \sqrt{ab} )^{2} + ( b - \sqrt{ab} )^{2} \bigr) + 2 ( 1 - u ) ( a - b )^{2}, \end{aligned}$$

*where* $h = \frac{b}{a}$, $r = \min \{ 2u,1 - 2u \}$ *and* $R = \max \{ 1 - 2r,2r \}$.

### Proof

It is to mention that the process always has the similarity due to the properties of the problem. So we only need to investigate the first inequality. By inequality (2.9), we have

$$\begin{aligned}& ( a + b )^{2} - K ( \sqrt{h},2 )^{R} \bigl( a^{1 - u}b^{u} + a^{u}b^{1 - u} \bigr)^{2} \\& \quad = a^{2} + b^{2} + 2ab - K ( \sqrt{h},2 )^{R} \bigl( a^{1 - u}b^{u} + a^{u}b^{1 - u} \bigr)^{2} \\& \quad = ( 1 - u )a^{2} + ub^{2} - K ( \sqrt{h},2 )^{R} \bigl( a^{1 - u}b^{u} \bigr)^{2} \\& \qquad {}+ ua^{2} + ( 1 - u )b^{2} - K ( \sqrt{h},2 )^{R} \bigl( a^{u}b^{1 - u} \bigr)^{2} + \bigl( 1 - K ( \sqrt{h},2 )^{R} \bigr)2ab \\& \quad \le r ( a - \sqrt{ab} )^{2} + u ( a - b )^{2} + r ( b - \sqrt{ab} )^{2} + u ( a - b )^{2}. \end{aligned}$$

Then we can conclude the inequality

$$( a + b )^{2} \le K ( \sqrt{h},2 )^{R} \bigl( a^{1 - u}b^{u} + a^{u}b^{1 - u} \bigr)^{2} + r \bigl( ( a - \sqrt{ab} )^{2} + ( b - \sqrt{ab} )^{2} \bigr) + 2u ( a - b )^{2}. $$

So the proof is completed. □

## New operator versions of Young-type inequalities

In the section, we will give some more excellent versions of Young-type operator inequalities and their reverse by the monotonic property of operator functions.

First, we present the monotonic property of operator function, which is the basis of the following discussion.

### Lemma 3.1

[13]

*Let* $T \in B ( H )$ *be self*-*adjoint*. *If* *f* *and* *g* *are both continuous functions with* $f ( t ) \ge g ( t )$ *for* $t \in \operatorname{Sp} ( T )$ (*where the sign* $\operatorname{Sp} ( T )$ *denotes the spectrum of operator* *T*), *then* $f ( T ) \ge g ( T )$.

Next we present our main results on the basis of inequalities (2.1)-(2.10). By Lemma 3.1, we have the following.

### Theorem 3.1

*Let* $A,B \in B ( H )$ *be positive invertible operators*, *I* *is the identity operator and* $u \in ( 0,1 )$. *If all positive numbers* *m*, $m'$ *and* *M*, $M'$ *satisfy either of the conditions* $0 < mI \le A \le m'I < M'I \le B \le MI$ *or* $0 < mI \le B \le m'I < M'I \le A \le MI$, *then*:

1. *If* $0 < {u} < \frac{1}{4}$, *then*
  3.1 $$\begin{aligned} A\nabla_{u}B \ge&2u ( A\nabla B - A\# B ) + 2u ( A\# B + A - 2A \#_{\frac{1}{4}}B ) \\ &{}+ r ( A + A\#_{\frac{1}{4}}B - 2A\#_{\frac{1}{8}}B ) + K \bigl( \sqrt[8]{h},2 \bigr)^{r_{1}}A\#_{u}B, \end{aligned}$$
  *where* $h = \frac{M}{m}$, $r = \min \{ 1 - 4u,4u \}$, $r_{1} = \min \{ 2r,1 - 2r \}$.
2. *If* $\frac{1}{4} \le u < \frac{1}{2}$, *then*
  3.2 $$\begin{aligned} A\nabla_{u}B \ge&2u ( A\nabla B - A\# B ) + ( 1 - 2u ) ( A\# B + A - 2A\#_{\frac{1}{4}}B ) \\ &{}+ r ( A\# B + A\#_{\frac{1}{4}}B - 2A\#_{\frac{3}{8}}B ) + K \bigl( \sqrt[8]{h},2 \bigr)^{r_{1}}A\#_{u}B, \end{aligned}$$
  *where* $h = \frac{M}{m}$, $r = \min \{ 2 - 4u,4u - 1 \}$, $r_{1} = \min \{ 2r,1 - 2r \}$.
3. *If* $\frac{1}{2} \le u < \frac{3}{4}$, *then*
  3.3 $$\begin{aligned} A\nabla_{u}B \ge&2 ( 1 - u ) ( A\nabla B - A\# B ) + ( 2u - 1 ) ( A\# B + B - 2A\#_{\frac{1}{4}}B ) \\ &{}+ r ( A\# B + A\#_{\frac{3}{4}}B - 2A\#_{\frac{5}{8}}B ) + K \bigl( \sqrt[8]{h},2 \bigr)^{r_{1}}A\#_{u}B, \end{aligned}$$
  *where* $h = \frac{M}{m}$, $r = \min \{ 3 - 4u,4u - 2 \}$, $r_{1} = \min \{ 2r,1 - 2r \}$.
4. *If* $\frac{3}{4} \le u < 1$, *then*
  3.4 $$\begin{aligned} A\nabla_{u}B \ge&2 ( 1 - u ) ( A\nabla B - A\# B ) + ( 2 - 2u ) ( A\# B + B - 2A\#_{\frac{1}{4}}B ) \\ &{}+ r ( B + A\#_{\frac{3}{4}}B - 2A\#_{\frac{7}{8}}B ) + K \bigl( \sqrt[8]{h},2 \bigr)^{r_{1}}A\#_{u}B, \end{aligned}$$
  *where* $h = \frac{M}{m}$, $r = \min \{ 4 - 4u,4u - 3 \}$, $r_{1} = \min \{ 2r,1 - 2r \}$.

*And these equalities hold if and only if* $A = B$ *and* $m = M$.

### Proof

We only need to investigate inequality (3.1) due to the similarity of the process of proof.

If $0 < {u} < \frac{1}{4}$, by inequality (2.1), for any $x > 0$, we have

$$( 1 - u ) + ux \ge u ( 1 - \sqrt{x} )^{2} + 2u \bigl( \sqrt[4]{x} - 1 \bigr)^{2} + r \bigl( 1 - \sqrt[8]{x} \bigr)^{2} + K \bigl( \sqrt[8]{x},2 \bigr)^{r_{1}}x^{u}. $$

For $X = A^{ - \frac{1}{2}}BA^{ - \frac{1}{2}}$, under the first condition, we get $I \le hI = \frac{M}{m}I \le X \le h'I = \frac{M'}{m'}$, and then $\operatorname{Sp} ( X ) \subseteq [ h,h' ] \subseteq ( 1, +\infty )$.

By Lemma 3.1, we have

$$\begin{aligned} ( 1 - u )I + uX \ge& u \bigl( I - 2X^{\frac{1}{2}} + X \bigr) + 2u \bigl( X^{\frac{1}{2}} - 2X^{\frac{1}{4}} + I \bigr) + r \bigl( I - 2X^{\frac{1}{8}} + X^{\frac{1}{4}} \bigr) \\ &{}+ \min_{h \le x \le h'}K \bigl( \sqrt[8]{x},2 \bigr)^{r_{1}}X^{u}. \end{aligned}$$

Since the Kantorovich constant $K ( t,2 ) = \frac{ ( 1 + t )^{2}}{4t}$ is an increasing function on $( 1, \infty )$, then

3.5 $$\begin{aligned} ( 1 - u )I + uA^{ - \frac{1}{2}}BA^{ - \frac{1}{2}} \ge& u \bigl( I - 2 \bigl( A^{ - \frac{1}{2}}BA^{ - \frac{1}{2}} \bigr)^{{\frac{1}{2}}} + A^{ - \frac{1}{2}}BA^{ - \frac{1}{2}} \bigr) \\ &{}+ 2u \bigl( \bigl( A^{ - \frac{1}{2}}BA^{ - \frac{1}{2}} \bigr)^{\frac{1}{2}} - 2 \bigl( A^{ - \frac{1}{2}}BA^{ - \frac{1}{2}} \bigr)^{\frac{1}{4}} + I \bigr) \\ &{}+r \bigl( I - 2 \bigl( A^{ - \frac{1}{2}}BA^{ - \frac{1}{2}} \bigr)^{\frac{1}{8}} + \bigl( A^{ - \frac{1}{2}}BA^{ - \frac{1}{2}} \bigr)^{\frac{1}{4}} \bigr) \\ &{}+ K \bigl( \sqrt[4]{h},2 \bigr)^{r_{1}} \bigl( A^{ - \frac{1}{2}}BA^{ - \frac{1}{2}} \bigr)^{u}. \end{aligned}$$

In a similar way, under the second condition, we have $I \le\frac{1}{h}I = \frac{m}{M}I \le X \le\frac{1}{h'}I = \frac{m'}{M'}$, and $\operatorname{Sp} ( X ) \subseteq [ \frac{1}{h},\frac{1}{h'} ] \subseteq ( 0,1 )$. By Lemma 3.1, we have

$$\begin{aligned} ( 1 - u )I + uX \ge& u \bigl( I - 2X^{\frac{1}{2}} + X \bigr) + 2u \bigl( X^{\frac{1}{2}} - 2X^{\frac{1}{4}} + I \bigr) + r_{0} \bigl( I - 2X^{\frac{1}{8}} + X^{\frac{1}{4}} \bigr) \\ &{}+ \min_{\frac{1}{h'} \le x \le\frac {1}{h}}K \bigl( \sqrt[8]{x},2 \bigr)^{r_{1}}X^{u}. \end{aligned}$$

Since the Kantorovich constant $K ( t,2 ) = \frac{ ( 1+ t )^{2}}{4t}$ is a decreasing function on $( 0,1 )$, then

3.6 $$\begin{aligned} ( 1 - u )I + uA^{ - \frac{1}{2}}BA^{ - \frac{1}{2}} \ge& u \bigl( I - 2 \bigl( A^{ - \frac{1}{2}}BA^{ - \frac{1}{2}} \bigr)^{{\frac{1}{2}}} + A^{ - \frac{1}{2}}BA^{ - \frac{1}{2}} \bigr) \\ &{}+ 2u \bigl( \bigl( A^{ - \frac{1}{2}}BA^{ - \frac{1}{2}} \bigr)^{\frac{1}{2}} - 2 \bigl( A^{ - \frac{1}{2}}BA^{ - \frac{1}{2}} \bigr)^{\frac{1}{4}} + I \bigr) \\ &{}+r_{0} \bigl( I - 2 \bigl( A^{ - \frac{1}{2}}BA^{ - \frac{1}{2}} \bigr)^{\frac{1}{8}} + \bigl( A^{ - \frac{1}{2}}BA^{ - \frac{1}{2}} \bigr)^{\frac{1}{4}} \bigr) \\ &{}+ K \biggl( \sqrt[8]{\frac{1}{h}},2 \biggr)^{r_{1}} \bigl( A^{ - \frac{1}{2}}BA^{ - \frac{1}{2}} \bigr)^{u}. \end{aligned}$$

Then multiplying inequalities (3.5) and (3.6) by $A^{\frac{1}{2}}$ on the left-hand side and on the right-hand side, we can deduce the required inequality (3.1). □

### Theorem 3.2

*Let* $A,B \in B ( H )$ *be positive invertible operators*, *I* *is the identity operator and* $u \in ( 0,1 )$. *If all positive numbers* *m*, $m'$ *and* *M*, $M'$ *satisfy either of the conditions* $0 < mI \le A \le m'I < M'I \le B \le MI$ *or* $0 < mI \le B \le m'I < M'I \le A \le MI$, *then*:

1. *If* $0 < {u} < \frac{1}{4}$, *then*
  3.7 $$\begin{aligned} A\nabla_{u}B \le&2 ( 1 - u ) ( A\nabla B - A\# B ) - 2u ( A\# B + B - 2A\#_{\frac{1}{4}}B ) \\ &{}- r ( B + A\#_{\frac{3}{4}}B - 2A\#_{\frac{7}{8}}B ) + K \bigl( \sqrt[8]{h},2 \bigr)^{ - r_{1}}A\#_{u}B, \end{aligned}$$
  *where* $h = \frac{M}{m}$, $r = \min \{ 1 - 4u,4u \}$, $r_{1} = \min \{ 2r,1 - 2r \}$.
2. *If* $\frac{1}{4} \le u < \frac{1}{2}$, *then*
  3.8 $$\begin{aligned} A\nabla_{u}B \le&2 ( 1 - u ) ( A\nabla B - A\# B ) - ( 1 - 2u ) ( A\# B + B - 2A\#_{\frac{1}{4}}B ) \\ &{}- r ( A\# B + A\#_{\frac{3}{4}}B - 2A\#_{\frac{5}{8}}B ) + K \bigl( \sqrt[8]{h},2 \bigr)^{ - r_{1}}A\#_{u}B, \end{aligned}$$
  *where* $h = \frac{M}{m}$, $r = \min \{ 2 - 4u,4u - 1 \}$, $r_{1} = \min \{ 2r,1 - 2r \}$.
3. *If* $\frac{1}{2} \le u < \frac{3}{4}$, *then*
  3.9 $$\begin{aligned} A\nabla_{u}B \le&2u ( A\nabla B - A\# B ) - ( 2u - 1 ) ( A\# B + A - 2A\#_{\frac{1}{4}}B ) \\ &{}- r ( A\# B + A\#_{\frac{1}{4}}B - 2A\#_{\frac{3}{8}}B ) + K \bigl( \sqrt[8]{h},2 \bigr)^{ - r_{1}}A\#_{u}B, \end{aligned}$$
  *where* $h = \frac{M}{m}$, $r = \min \{ 3 - 4u,4u - 2 \}$, $r_{1} = \min \{ 2r,1 - 2r \}$.
4. *If* $\frac{3}{4} \le u < 1$, *then*
  3.10 $$\begin{aligned} A\nabla_{u}B \le&2u ( A\nabla B - A\# B ) - ( 2 - 2u ) ( A\# B + A - 2A\#_{\frac{1}{4}}B ) \\ &{}- r ( A + A\#_{\frac{1}{4}}B - 2A\#_{\frac{1}{8}}B ) + K \bigl( \sqrt[8]{h},2 \bigr)^{ - r_{1}}A\#_{u}B, \end{aligned}$$
  *where* $h = \frac{M}{m}$, $r = \min \{ 4 - 4u,4u - 3 \}$, $r_{1} = \min \{ 2r,1 - 2r \}$.

*And these equalities hold if and only if* $A = B$ *and* $m = M$.

### Proof

The process of the proof is analogous to that of Theorem 3.1, so we omit it here. □

### Theorem 3.3

*Let* $A,B \in B ( H )$ *be positive invertible operators*, *I* *is the identity operator and* $u \in ( 0,1 )$. *If all positive numbers* *m*, $m'$ *and* *M*, $M'$ *satisfy either of the conditions* $0 < mI \le A \le m'I < M'I \le B \le MI$ *or* $0 < mI \le B \le m'I < M'I \le A \le MI$, *then*:

1. *If* $0 < u < \frac{1}{2}$, *then*
  $$A\nabla B \le2u ( A\nabla B - A\# B ) + r ( A\# B + A - 2A\#_{\frac{1}{4}}B ) + K ( \sqrt{h},2 )^{R}A\#_{u}B; $$
2. *If* $\frac{1}{2} \le u < 1$, *then*
  $$A\nabla B \le2 ( 1 - u ) ( A\nabla B - A\# B ) + r ( A\# B + B - 2A\#_{\frac{1}{4}}B ) + K ( \sqrt{h},2 )^{R}A\#_{u}B, $$

*where* $r = \min \{ 2u,1 - 2u \}$, $R = \max \{ 2r,1 - 2r \}$ *and* $h = \frac{M}{m}$.

### Proof

We only need to investigate the first inequality due to the similarity of the process of proof.

By inequality (2.9), for any $x > 0$, we have

$$( 1 - u ) + ux \le u ( 1 - \sqrt{x} )^{2} + r \bigl( 1 - \sqrt[4]{x} \bigr)^{2} + K ( \sqrt{h},2 )^{R}x^{u}. $$

For $X = A^{ - \frac{1}{2}}BA^{ - \frac{1}{2}}$, under the first condition, we get $I \le hI = \frac{M}{m}I \le X \le h'I = \frac{M'}{m'}$.

By Lemma 3.1 and the property of the Kantorovich constant, we have

$$\begin{aligned} ( 1 - u )I + uA^{ - \frac{1}{2}}BA^{ - \frac{1}{2}} \le& u \bigl( I - \bigl( A^{ - \frac{1}{2}}BA^{ - \frac{1}{2}} \bigr)^{\frac{1}{2}} \bigr)^{2} + r \bigl( I - \bigl( A^{ - \frac{1}{2}}BA^{ - \frac{1}{2}} \bigr)^{\frac{1}{4}} \bigr)^{2} \\ &{}+ K ( \sqrt{h},2 )^{R} \bigl( A^{ - \frac{1}{2}}BA^{ - \frac{1}{2}} \bigr)^{u}. \end{aligned}$$

Then multiplying the above inequality by $A^{\frac{1}{2}}$ on the left-hand side and right-hand side respectively, we can deduce the required inequality. The investigation under the other condition is similar to the above, so we omit it here. □

### Remark 3.1

We get the promotion of the inequality by Liao et al. in [8]. Since the Kantorovich constant $K ( t,2 ) = \frac { ( t + 1 )^{2}}{4t} > 1$ for $t > 0$ and ${t} \ne1$, combining (3.1) and (3.7) for $0 < u < \frac{1}{4}$, we have

$$\begin{aligned} 0 \le& A\#_{u}B \le2u ( A\nabla B - A\# B ) + A\#_{u}B \\ \le&2u ( A\nabla B - A\# B ) + 2u ( A\# B + A - 2A\#_{\frac{1}{4}}B ) + r ( A + A\#_{\frac{1}{4}}B - 2A\#_{\frac{1}{8}}B ) \\ \le&2u ( A\nabla B - A\# B ) + 2u ( A\# B + A - 2A\#_{\frac{1}{4}}B ) \\ &{}+ r ( A + A\#_{\frac{1}{4}}B - 2A\#_{\frac{1}{8}}B ) + K \bigl( \sqrt[8]{h},2 \bigr)^{r_{1}}A\#_{u}B \\ \le& A\nabla_{u}B \le2 ( 1 - u ) ( A\nabla B - A\# B ) - 2u ( A\# B + B - 2A\#_{\frac{1}{4}}B ) \\ &{}- r ( B + A\#_{\frac{3}{4}}B - 2A\#_{\frac{7}{8}}B ) + K \bigl( \sqrt[8]{h},2 \bigr)^{ - r_{1}}A\#_{u}B \\ \le&2 ( 1 - u ) ( A\nabla B - A\# B ) - r ( B + A\#_{\frac{3}{4}}B - 2A \#_{\frac{7}{8}}B ) + K \bigl( \sqrt[8]{h},2 \bigr)^{ - r_{1}}A \#_{u}B \\ \le&2 ( 1 - u ) ( A\nabla B - A\# B ) - r ( B + A\#_{\frac{3}{4}}B - 2A \#_{\frac{7}{8}}B ) + A\#_{u}B \\ \le&2 ( 1 - u ) ( A\nabla B - A\# B ) + A\#_{u}B. \end{aligned}$$

A similar analysis can be performed in other cases, so we omit them here.

## New matrix versions of Young’s inequalities for the Hilbert-Schmidt norm

In the last part, we focus on the matrix version of Young’s inequality for the Hilbert-Schmidt norm. As is well known, every positive semi-definite matrix can be unitarily diagonalizable. Then, for any positive semi-definite matrices *A* and *B*, there exist two unitary matrices *U* and *V* such that $A = U\operatorname{diag} ( \lambda_{1},\lambda_{2}, \ldots,\lambda_{n} )U^{*}$ and $B = V\operatorname{diag} ( \mu_{1},\mu_{2}, \ldots,\mu_{n} )V^{*}$ ($\lambda_{i} \ge0$, $\mu_{i} \ge0$, $i = 1,2,\ldots,n$). The spectrum of *A* and *B* is denoted by $\operatorname{Sp} ( A ) = \{ \lambda_{1},\lambda_{2},\ldots,\lambda_{n} \}$ and $\operatorname{Sp} ( B ) = \{ \mu_{1},\mu_{2}, \ldots,\mu_{n} \}$, respectively.

### Theorem 4.1

*Suppose* $A,B,X \in M_{n}$ *and* *A*, *B* *are positive semi*-*definite matrices*, *I* *is the identity operator*, *and* $0 < mI \le A,B \le MI$ *and* $u \in ( 0,1 )$. *Then we have the following*:

*If* $0 < u < \frac{1}{2}$, *then*

$$\begin{aligned} \begin{aligned} \bigl\Vert ( 1 - u )AX + uXB \bigr\Vert _{2}^{2} \le{}& u^{2} \Vert AX - XB \Vert _{2}^{2} + r \bigl\Vert AX - A^{\frac{1}{2}}XB^{\frac{1}{2}} \bigr\Vert _{2}^{2} \\ &{}+ K ( \sqrt{h},2 )^{R} \bigl\Vert A^{1 - u}XB^{u} \bigr\Vert _{2}^{2}. \end{aligned} \end{aligned}$$

*If* $\frac{1}{2} \le u < 1$, *then*

$$\begin{aligned} \bigl\Vert ( 1 - u )AX + uXB \bigr\Vert _{2}^{2} \le& ( 1 - u )^{2} \Vert AX - XB \Vert _{2}^{2} + r \bigl\Vert XB - A^{\frac{1}{2}}XB^{\frac{1}{2}} \bigr\Vert _{2}^{2} \\ &{}+ K ( \sqrt{h},2 )^{R} \bigl\Vert A^{1 - u}XB^{u} \bigr\Vert _{2}^{2}, \end{aligned}$$

*where* $h = \frac{b}{a}$, $r = \min \{ 2u,1 - 2u \}$, $R = \max \{ 2r,1 - 2r \}$.

### Proof

The proof of the second inequality is similar to that of the first one. Thus we only need to investigate the first one. Since *A* and *B* are positive matrices, by the spectral decomposition theorem, there exist unitary matrices $U,V \in M_{n}$ satisfying $A = U\Lambda _{1}U^{*}$, $B = V\Lambda_{2}V^{*}$, where $\Lambda_{1} = \operatorname{diag} ( \lambda_{1},\lambda_{2}, \ldots,\lambda_{n} )$, $\Lambda_{2} = \operatorname{diag} ( \mu_{1},\mu_{2}, \ldots,\mu_{n} )$ ($\lambda_{i} \ge0$, $\mu_{i} \ge0$, $i = 1,2,\ldots,n$).

Suppose $Y = U^{*}XV = [y_{ij}]$, we have

$$\begin{aligned}& ( 1 - u )AX + uXB = U \bigl( ( 1 - u )\Lambda_{1}Y + \mu Y \Lambda_{2} \bigr)V^{*} = U\bigl[ \bigl( ( 1 - u )\lambda _{i} + u\mu_{j} \bigr)y_{ij}\bigr]V^{*}, \\& AX - XB = U\bigl[ ( \lambda_{i} - \mu_{j} )y_{ij}\bigr]V^{*},\quad \mbox{and}\quad A^{1 - u}XB^{u} = U \bigl( \lambda_{i}^{1 - u} \mu_{j}^{u}y_{ij} \bigr)V^{*}. \end{aligned}$$

By Theorem 2.4 and the unitarily invariant property of the Hilbert-Schmidt norm, we can see that

$$\begin{aligned}& \bigl\Vert ( 1 - u )AX + uXB \bigr\Vert _{2}^{2} \\& \quad = \sum_{i,j = 1}^{n} \bigl( ( 1 - u ) \lambda_{i} + u\mu_{j} \bigr)^{2} \vert y_{ij} \vert ^{2} \\& \quad \le\sum_{i,j = 1}^{n} \bigl( \max K ( \sqrt{t_{ij}},2 )^{R} \bigl( \lambda_{i}^{2 - 2u} \mu_{j}^{2u} \bigr) + u^{2} ( \lambda_{i} - \mu_{j} )^{2} + r ( \lambda_{i} - \sqrt{ \lambda_{i}\mu_{j}} )^{2} \bigr) \vert y_{ij} \vert ^{2}, \end{aligned}$$

where $t_{ij} = \frac{\lambda_{i}}{\mu_{j}}$.

Utilizing the condition $0 < mI \le A,B \le MI$, $\frac{m}{M} = \frac{1}{h} \le t_{ij} = \frac{\lambda_{i}}{\mu_{j}} \le h = \frac{M}{m}$ and the property of the Kantorovich constant, we have

$$\begin{aligned}& \bigl\Vert ( 1 - u )AX + uXB \bigr\Vert _{2}^{2} \\& \quad \le \sum_{i,j = 1}^{n} \bigl( K ( \sqrt{h},2 )^{R} \bigl( \lambda_{i}^{2 - 2u} \mu_{j}^{2u} \bigr) + u^{2} ( \lambda_{i} - \mu_{j} )^{2} + r ( \lambda_{i} - \sqrt{ \lambda_{i}\mu_{j}} )^{2} \bigr) \vert y_{ij} \vert ^{2} \\& \quad = K ( \sqrt{h},2 )^{R}\sum_{i.j = 1}^{n} \bigl( \lambda _{i}^{2 - 2u}\mu_{j}^{2u} \bigr) \vert y_{ij} \vert ^{2} + u^{2}\sum _{i,j = 1}^{n} ( \lambda_{i} - \mu_{j} )^{2} \vert y_{ij} \vert ^{2} + r\sum_{i,j = 1}^{n} ( \lambda_{i} - \sqrt{\lambda_{i}\mu_{j}} )^{2} \vert y_{ij} \vert ^{2} \\& \quad = K ( \sqrt{h},2 )^{R} \bigl\Vert A^{1 - u}XB^{u} \bigr\Vert _{2}^{2} + u^{2} \Vert AX - XB \Vert _{2}^{2} + r \bigl\Vert AX - A^{\frac{1}{2}}XB^{\frac{1}{2}} \bigr\Vert _{2}^{2}. \end{aligned}$$

So the proof is completed. □

### Theorem 4.2

*Let* $A,B \in M_{n}$ *such that* *A*, *B* *are positive invertible operators*, *I* *is the identity operator*, $0 < mI \le A,B \le MI$ *and* $u \in ( 0,1 )$. *We have the following*:

*If* $0 < u < \frac{1}{2}$, *then*

$$\begin{aligned} \Vert AX + XB \Vert _{2}^{2} \le& r \bigl( \bigl\Vert AX - A^{\frac{1}{2}}XB^{\frac{1}{2}} \bigr\Vert _{2}^{2} + \bigl\Vert XB - A^{\frac{1}{2}}XB^{\frac{1}{2}} \bigr\Vert _{2}^{2} \bigr) + 2u \Vert AX - XB \Vert _{2}^{2} \\ &{}+ K ( \sqrt{h},2 )^{R} \bigl\Vert A^{1 - u}XB^{u} + A^{u}XB^{1 - u} \bigr\Vert _{2}^{2}. \end{aligned}$$

*If* $\frac{1}{2} \le u < 1$, *then*

$$\begin{aligned} \Vert AX + XB \Vert _{2}^{2} \le& r \bigl( \bigl\Vert AX - A^{\frac{1}{2}}XB^{\frac{1}{2}} \bigr\Vert _{2}^{2} + \bigl\Vert XB - A^{\frac{1}{2}}XB^{\frac{1}{2}} \bigr\Vert _{2}^{2} \bigr) + 2 ( 1 - u ) \Vert AX - XB \Vert _{2}^{2} \\ &{}+ K ( \sqrt{h},2 )^{R} \bigl\Vert A^{1 - u}XB^{u} + A^{u}XB^{1 - u} \bigr\Vert _{2}^{2}, \end{aligned}$$

*where* $h = \frac{M}{m}$, $r = \min \{ 2u,1 - 2u \}$, $R = \min \{2r,1 - 2r \}$.

### Proof

The proof of the second inequality is similar to that of the first one. Thus we only need to investigate the first one. Since *A* and *B* are positive matrices, by the spectral decomposition theorem, there exist unitary matrices $U,V \in M_{n}$ satisfying $A = U\Lambda _{1}U^{*}$, $B = V\Lambda_{2}V^{*}$, where $\Lambda_{1} = \operatorname{diag} ( \lambda_{1},\lambda_{2}, \ldots,\lambda_{n} )$, $\Lambda_{2} = \operatorname{diag} ( \mu_{1},\mu_{2}, \ldots,\mu_{n} )$ ($\lambda_{i} \ge0$, $\mu_{i} \ge0$, $i = 1,2,\ldots,n$).

Suppose $Y = U^{*}XV = [y_{ij}]$, we have

$$\begin{aligned}& AX + XB = U\bigl[ ( \lambda_{i} + \mu_{j} )y_{ij}\bigr]V^{*},\qquad AX - XB = U\bigl[ ( \lambda_{i} - \mu_{j} )y_{ij}\bigr]V^{*},\quad\mbox{and} \\& A^{1 - u}XB^{u} + A^{u}XB^{1 - u} = U \bigl( \lambda_{i}^{1 - u}\mu _{j}^{u}y_{ij} + \lambda_{i}^{u}\mu_{j}^{1 - u}y_{ij} \bigr)V^{*}. \end{aligned}$$

And so

$$\bigl\Vert A^{1 - u}XB^{u} + A^{u}XB^{1 - u} \bigr\Vert _{2}^{2} = \sum_{i,j = 1}^{n} \bigl( \lambda_{i}^{1 - u}\mu_{j}^{u} + \lambda_{i}^{u}\mu_{j}^{1 - u} \bigr)^{2} \vert y_{ij} \vert ^{2}. $$

By Theorem 2.5 and the unitarily invariant property of the Hilbert-Schmidt norm, we can see that

$$\begin{aligned} \Vert AX + XB \Vert _{2}^{2} =& \sum _{i,j = 1}^{n} ( \lambda_{i} + \mu_{j} )^{2} \vert y_{ij} \vert ^{2} \\ \le&\sum_{i,j = 1}^{n} \bigl( r \bigl( ( \lambda_{i} - \sqrt{\lambda_{i}\mu_{j}} )^{2} + ( \mu_{j} - \sqrt{\lambda_{i} \mu_{j}} )^{2} \bigr) + 2u ( \lambda_{i} - \mu_{j} )^{2} \\ &{}+ \max K ( \sqrt{t_{ij}},2 )^{R} \bigl( \lambda_{i}^{1 - u}\mu_{j}^{u} + \lambda_{i}^{u}\mu_{j}^{1 - u} \bigr)^{2} \bigr) \vert y_{ij} \vert ^{2}, \end{aligned}$$

where $t_{ij} = \frac{\lambda_{i}}{\mu_{j}}$. Under the condition $0 < mI \le A,B \le MI$, $\frac{m}{M} = \frac{1}{h} \le t_{ij} = \frac{\lambda_{i}}{\mu_{j}} \le h = \frac{M}{m}$ and by the property of the Kantorovich constant, we have

$$\begin{aligned} \Vert AX + XB \Vert _{2}^{2} \le&\sum_{i,j = 1}^{n} \bigl( r \bigl( ( \lambda_{i} - \sqrt{\lambda_{i}\mu_{j}} )^{2} + ( \mu_{j} - \sqrt{\lambda_{i} \mu_{j}} )^{2} \bigr) + 2u ( \lambda_{i} - \mu_{j} )^{2} \\ &{}+ K ( \sqrt{h},2 )^{R} \bigl( \lambda _{i}^{1 - u}\mu_{j}^{u} + \lambda_{i}^{u}\mu_{j}^{1 - u} \bigr)^{2} \bigr) \vert y_{ij} \vert ^{2} \\ =& r\sum_{i,j = 1}^{n} \bigl( ( \lambda_{i} - \sqrt{\lambda_{i}\mu_{j}} )^{2} + ( \mu_{j} - \sqrt{\lambda_{i} \mu_{j}} )^{2} \bigr) \vert y_{ij} \vert ^{2} + 2u\sum_{i,j = 1}^{n} ( \lambda_{i} - \mu_{j} )^{2} \vert y_{ij} \vert ^{2} \\ & {}+ K ( \sqrt{h},2 )^{R}\sum_{i,j = 1}^{n} \bigl( \lambda _{i}^{1 - u}\mu_{j}^{u} + \lambda_{i}^{u}\mu_{j}^{1 - u} \bigr)^{2} \vert y_{ij} \vert ^{2} \\ =& r \bigl( \bigl\Vert AX - A^{\frac{1}{2}}XB^{\frac{1}{2}} \bigr\Vert _{2}^{2} + \bigl\Vert XB - A^{\frac{1}{2}}XB^{\frac{1}{2}} \bigr\Vert _{2}^{2} \bigr) + 2 u \Vert AX - XB \Vert _{2}^{2} \\ &{}+ K ( \sqrt{h},2 )^{R} \bigl\Vert A^{1 - u}XB^{u} + A^{u}XB^{1 - u} \bigr\Vert _{2}^{2}. \end{aligned}$$

So the proof is completed. □
